# Service robotics: an emergent technology field at the interface between industry and services

**DOI:** 10.1007/s10202-012-0110-9

**Published:** 2012-11-16

**Authors:** Ingrid Ott

**Affiliations:** 1 Karlsruhe Institute of Technology (KIT), Karlsruhe, Germany; 2 Kiel Institute for the World Economy (IfW), Kiel, Germany

## Abstract

The paper at hand analyzes the economic implications of service robots as expected important future technology. The considerations are embedded into global trends, focusing on the interdependencies between services and industry not only in the context of the provision of services but already starting at the level of the innovation process. It is argued that due to the various interdependencies combined with heterogenous application fields, the resulting implications need to be contextualized. Concerning the net labor market effects, it is reasonable to assume that the field of service robotics will generate overall job creation that goes along with increasing skill requirements demanded from involved employees. It is analyzed which challenges arise in evaluating and further developing the new technology field and some policy recommendations are given.

## Abstract (de)

Der Beitrag analysiert die ökonomischen Implikationen von Service-Robotik als künftig bedeutendes Technologiefeld. Die Überlegungen werden hierbei in die globalen Trends eingebunden, fokussieren auf die Interdependenzen zwischen Dienstleistung und Industrie, wobei es nicht nur um die Bereitstellung einer Dienstleistung an sich geht, sondern auch der Innovationsprozess einbezogen wird. Es wird argumentiert, dass die vielfältigen Interdependenzen verbunden mit den heterogenen Einsatzfeldern der Service-Roboter es erforderlich machen, dass die Aussagen kontextualisiert erfolgen. Insgesamt wird erwartet, dass vom Feld der Service-Robotik positive Arbeitsmarkteffekte ausgehen, wobei die Anforderungen an das Bildungsniveau der Beschäftigten steigen werden. Weiterhin wird analysiert, welche Herausforderungen zu bewältigen sind, wenn es darum geht, ein neues Technologiefeld zu bewerten und weiter zu entwickeln und es werden einige Politikimplikationen abgeleitet.

## Introduction

Service robots represent a promising future technology that lies at the interface between industry and service sector. Basically, a high technology is utilized to solve ongoing and future challenges, for example, demographic change, national security, public health or environmental protection. In this context, the development of information and communication technologies (ICT) in the last several decades plays a crucial role since, aside from mechanical features, programming and the underlying hardware are key components of service robots. Due to miniaturization, the necessary computing capacities are nowadays available at reasonable prices and may be implemented in the mechanical parts of the machine. This increasingly allows for individual and decentralized application areas with the consequence that robots nowadays are more and more utilized outside of industry, namely in the service sector or in personal life. Service robots in this context form part of the knowledge economy which unifies the knowledge-intensive parts of services and industry (compare Gehrke et al. 2010).

The underlying technology development is embedded in various trends among them ongoing growth in industrialized countries, demographic and sectoral change. In this context, the tertiary sector has undergone a shift from traditional to novel types of services. The latter are characterized by strong knowledge intensity that is mostly due to their linkages to certain technologies. At the same time, both industry/technology and services are increasingly becoming interdependent ranging from R&D over production to sales. The resulting value creation processes are hybrid in the sense that they combine the provision of a good or technology with a certain service.

The described development is driven both by demand-side and supply-side arguments. The latter are, for example, due to the aforementioned cost degressions in ICT but they are also the outcome of directed industrial policies. Firms are embedded in international and fragmented value creation chains while governments wish to support international competitiveness and welfare in the home country. Aside from direct interventions, the government also may impact the technology development by setting standards or if it appears as a big user. This appears like a guarantee for a certain level of demand thereby reducing uncertainty inherent in innovation processes at the firm level (e.g. Bresnahan 2010 or Grossman and Helpman 1991). Demand-side arguments in the context of service robots mainly arise from future trends; among them, demographic change requires more and also novel types of services.

The paper at hand analyzes the economic implications of service robots as expected important future technology. The considerations are embedded into global trends, focusing on the interdependencies between services and industry not only in the context of the provision of services but already starting at the level of the innovation process. We argue that due to the various interdependencies combined with heterogenous application fields, the resulting implications need to be contextualized. However, some general thoughts and policy recommendations are provided.

The remainder of the paper is as follows. After discussing the interdependencies between technology and services as well as their linkages to the knowledge economy in Sect. 2 and a detailed presentation of the recent market situation in Sect. 3, some future perspectives of service robots are discussed in Sect. 4. The paper closes with a short summary and derives some policy recommendations.

## High technologies and services

The structural change goes along with a strong increase in knowledge intensity both in the secondary sector (industry) and the tertiary sector (services). The latter has not only grown tremendously during the last several decades, but also has undergone a change in composition: In addition to traditional services, increasingly novel types of services have been created. These are mostly either based on new technologies or require their utilization. At the same time, technology oriented branches more and more adopt services within their firm strategies thereby covering the entire value creation process starting from research and development (R&D) until production. As a consequence (especially high), technology and (knowledge-intensive) services become increasingly interdependent. This artefact is reflected in the so-called knowledge economy that unifies the knowledge-intensive parts of the industry and service sector (compare, for example, Gehrke et al. 2010). Recent indicators to assign any economic activity to this field are the expenses in R&D (at least 2.5 % of total revenues) or highly skilled workers.

In order to better understand the role of technology within the knowledge economy, at first a closer look at *traditional services* may be helpful (e.g. Chesbrough and Spohrer 2006 or Maleri and Frietzsche 2008). Services may be distinguished from usual goods in various respects: they are experience goods, mostly immaterial and the quality only becomes obvious while utilizing the service provided. The simultaneity of production and consumption and correspondingly the direct link between service provider and beneficiary imply that the service may not be stored, changed or sold again. For the service provider, costs are sunk and non-reversible. Due to the difficulty in storage, supply and demand have to be chronologically coordinated and usually providers locate close by their clients. Due to human interaction, the possibilities of standardization are limited.

As argued before, especially, the economic importance of *knowledge-intensive services* has continuously increased. Today, already one-third of the employees and a large share of self-employed persons are active in this branch (compare for Germany, for example, http://www.bmwi.de/DE/Themen/Wirtschaft/dienstleistungswirtschaft.html).

**Fig. 1:**
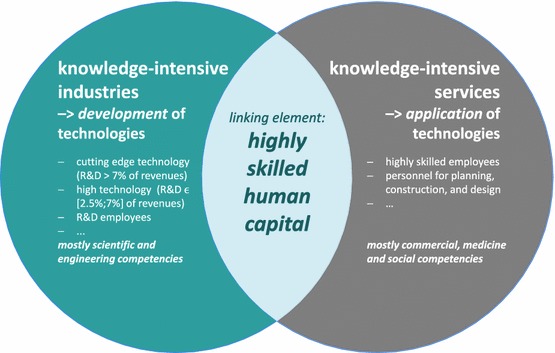
Features of the knowledge economy: development and application of technologies; own presentation

Figure 1 provides a stylized representation of the knowledge economy. Linking element between the two knowledge-intensive fields (namely, services and industries) are highly skilled workers that are confronted with different skill requirements. Within industry, workers are mainly involved in developing technologies thereby utilizing technological skills whereas in the field of services mostly technology application is the central feature which requires skills beyond pure technological knowledge including design, management or social competencies.

As in any field, innovation (or technological progress) is also the key driver of productivity gains in the knowledge economy. The crucial point here is a circular relationship between knowledge-intensive services and industries in the following sense: frequently, the economic success of a technology depends upon the parallel development of an accompanying innovative service. In this context, services then determine the direction in which innovative activity of the technology takes place. At the same time, modern technologies increase the quality of certain services or even enable their provision. Innovation processes in knowledge-intensive services and industries are thus complementary and result in hybrid innovation and value creation processes.

## Service robotics: some stylized facts

Service robotics is an emerging technology field that utilizes a high technology in order to provide services. As such, a unique assignment to the official industry classification or any national account system does not yet exist. This impedes a comprehensive assessment of the economic importance of service robotics with respect to employment, contribution to aggregate value creation or innovation activities. Since 2007, an international study group has been re-working the European robotics norm ISO 8373 in order to add an official definition of service robots (compare the homepage of the International Federation of Robotics (IFR); www.ifr.org). In the meantime, the following unifying characteristics are broadly accepted: service robots are utilized outside of factories and contribute to both traditional and new type services. 1 To systematize the operational areas, the IFR provides the following working definition: 2

A service robot is a robot which operates semi- or fully autonomously to perform services useful to the well being of humans and equipment, excluding manufacturing operations.

**Fig. 2:**
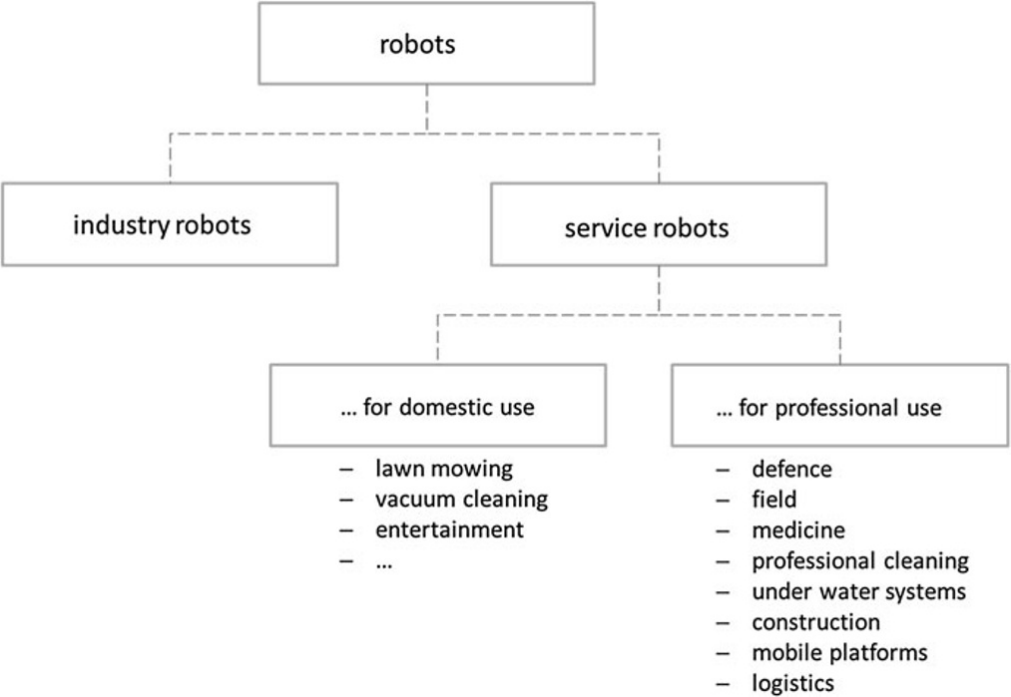
Utilization of service robots; own representation according to the classification of the IFR

Figure 2 outlines industrial and service robots and illustrates the operational areas. Recent estimations of the IFR stress the economic predominance of professional over private robotic systems. 3 This estimation is based on the sales realized during the last decade but also confirmed by the forecasts of market potentials in the coming years. Market data of the year 2011 report almost 16,000 sold units of professional service robots and corresponding revenues of 3.6 bn US$ (compare Table 1). 4 It becomes obvious that there is a strong concentration in only three application areas, namely defence, field and medicine, that account for almost 80 % of sold units and even 88 % of all revenues generated. These areas are heterogenous in several respects as might be seen by taking a closer look at selected characteristics (labor market implications, relation to subordinate governmental tasks, human--machine interaction): 5

- Defence, especially unmanned aerial vehicle/drone:

Governmental demand provides the basis for large amounts of service robots in the field of defence. This brings about planning reliability for the firms that are active in the corresponding production but also in research and development. Due to large amounts produced, firms are able to realize learning effects thereby lowering production costs and decreasing unit prices. Already nowadays drones are also utilized outside the fields of defence, for example, by television stations that report on natural disasters in regions that are not accessible via traditional infrastructure.

- Agriculture and field, especially milking robots:

Labor-augmenting technological change has led to large productivity increases in agriculture since the industrial revolution. This trend is reinforced by the use of service robots especially in large-scale operations that are close to industrial production. In this context, the robot substitutes low-skill working places.

- Medicine, especially surgery assistance:

Here, the service robot complements the working activity of highly skilled employees with the goal to reduce costs and/or treatment mistakes. Besides, service robots become increasingly important in professional and home care, for example, in the context of lifting people or transporting medicine or laundry. Due to the demographic change, this field is expected to grow continuously and service robots shall help to reduce skills shortage.

**Table 1:** Worldwide sales of service robots in 2011; own presentation and calculations based on the IFR press release, September 2012

Category	Units		Total revenue	
	Absolute	Relative	Absolute	Relative
			(Mio US$)	
Defence	6,570	40.0 %	722.70	20.1 %
Field robots	5,087	31.0 %	915.66	25.4 %
Medical robots	1,051	6.4 %	1,534.66	42.6 %
⋮				
∑ Others	3,700	22.6 %	427.18	11.9 %
Cumulated	16,408	1	3,600	1

**Fig. 3:**
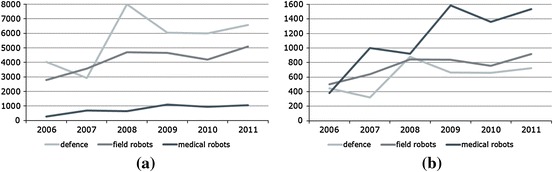
Sold units and generated revenues in the three most important application areas of professional service robots; own calculation and presentation based on information of the annual press releases of the IFR 2007–2012. The calculations in **b** assume the following average unit prices: field 0.18 Mio US$ defence 0.11 Mio US$ medicine 1.46 Mio US$. **a** Sold units, **b** generated revenues

Figure 3 confirms the mentioned composition in a dynamic perspective where defence dominates with respect to sold units, whereas medical robots, due to their high unit prices, provide the highest revenues. Besides, a closer look at the data highlights that all three fields continuously grow thereby keeping their relative importance almost stable across time.

The dominance of the three mentioned areas is probably based on very different mechanisms: especially with respect to the so-called superordinate governmental duties and responsibilities, the government provides continuous demand as well as planning reliability for the firms thereby affecting the underlying innovation processes (defence). An analogous effect may be identified by the demographic change for the growing health care market (medicine). Agriculture and field robots, in contrast, seem to be close to traditional industry robots thereby just providing an incremental advancement of the technology by implementing it to a new environment outside a usual factory. However, the short sketch of the mentioned three fields already indicates that the economic implications crucially depend upon the context. Due to the strong heterogeneity of the operational areas and the according mechanisms, a general estimation of the economic implications of service robotics is thus impossible but one has to carefully have in mind the respective peculiarities in order to derive policy recommendations.

## Some perspectives of service robots . . . 

### **… as an emergent technology field**

As already argued, service robotics is at the interface between industry and service sector. So far it is neither part of any existing official industry, patent or trademark classification system nor of any concordances that allow to frame the technology field or to estimate its economic implications immediately. 6 The mentioned databases are necessarily always delayed compared with the technological state of the art. At the same time, especially high technologies and expected future industries are in the core focus of national innovation policies. For being effective and efficient, these policies strongly rely on credible databases that include the entire value creation chains, starting from research and development over production and sales. Thus, the quality of the underlying databases is of major importance. Wrong assumptions or bad data have distortionary effects with an overall negative impact at an aggregate economic level. 7

### **… and innovation**

The further development of service robots takes place in the arc of suspense between supply (technology push) and demand (demand pull). 8 Thereby, especially the interdependencies between services on the one hand and technology on the other hand have to be considered. The supply side has strongly benefited from ongoing miniaturization and progress in the field of ICT. Demand-side arguments are mainly based on future trends, among them the demographic change. Besides, high technologies are frequently in the focus of governmental support while at the same time the actors are involved in an international technology competition. Governmental interventions in the market process pursue the goal to strengthen international competitiveness, to setup lead markets and/or to support the technological advances of the home country to generate long-term income and welfare. Due to ongoing globalization processes, the corresponding profits are increasingly generated in international markets. Besides, the government affects innovation activities by setting the legal framework for the firm’s activity: for example, in the field of public health care, the government decides which features are reimbursed by the health insurance. This influences individual demand for health services thereby also affecting medical supply.

Due to different legislations, the steering effect of national policies may differ between countries. This may also indirectly impact technologies as can be seen, for example, in the context of software patenting that is allowed in the US but not possible within the EU. Since software is an important component of service robotics, the different national patent legislations may distort the technology development internationally. Another argument by connecting services and technology lies in standardization. Its implications are not restricted to the technical dimension but are also crucial with respect to service exports and the exploitation of new markets.

### **… and human capital**

**Fig. 4:**
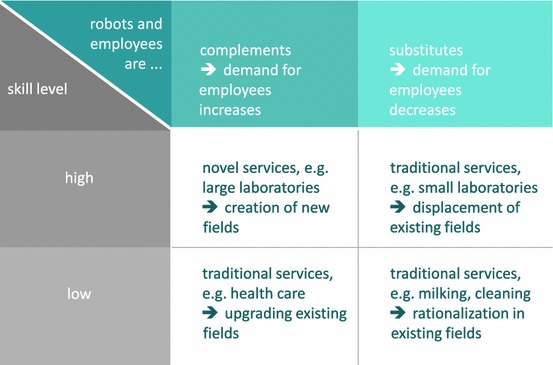
Labor market effects in the context of the application of service robots; own presentation

It is reasonable to assume that the number of highly qualified positions will increase as the technology field of service robots grows. This is expected to be true concerning both development and application of the technology. However, with respect to technology application, the discussion is a bit more sophisticated (compare also Fig. 4): concerning low-skill job positions, a service robot may basically substitute for the human employee as, for example, argued in the context of the milking robot. If, in contrast, the service robots complement human work force, positive labor market effects are probable independent of the workers’ qualification level. The service robot does not only provide new jobs in the context of novel services, but also it may enhance so far unattractive working conditions, for example, by facilitating physically exhausting activities, by creating free space for the provision of the original service or by valorizing the reputation of certain vocational fields due to the use of the high technology.

Altogether, the mentioned effects tend to result in a substitution of only lowly skilled working places by robots. Substitution will especially *not* take place if the service robots complement human activity. Concerning the net labor market effects of both technology development and application it is reasonable to assume that service robots end up in overall job creation that goes along with increasing skill requirements requested from the involved employees.

## Conclusions

Service robots represent a high technology that is utilized to provide certain services thereby mostly adding to the work of persons. Only seldom the service robots provide the service on their own but then the activity is at least surveyed by somebody at a (sometimes remote) control unit. The application areas of service robots are very heterogenous which makes it difficult to derive general statements concerning their economic implications. However, the following reasonings seem to be quite plausible.

The observed increase in the entire field of service robots is strongly driven by technology development in the field of ICT. Especially important here are miniaturization, cost degression and increased computing capacity which allows to implement hard- and software components in the mechanical parts of the robot.

The strong interdependency between technology and services which seems to be a characterizing element of the knowledge economy traces back to the innovation processes. Better technology allows for more application areas. At the same time, the technology development is driven by the needs of consumers and their demand for specific services. Here, especially the demographic change seems to become a crucial determinant of future technology development.

Overall, the field of service robots contributes to job creation which is clear in the context of technology development. A positive net effect is also reasonable when applying the technology although the use of service robots requires certain skills at the level of the employees. Overall the skill intensity will increase and lowly skilled working places will continuously be substituted by specialists.

Especially in the context of high technologies, the probability of governmental intervention in the process of technology development is quite high. The underlying arguments are manifold. The government acts as a concerted big demand thereby reducing uncertainty at the firm level. Various promotional programs have been set up at national and supranational levels (e.g. http://www.robotics-platform.eu/cms/index.php or www.service-robotik-initiative.de/). The involved actors are embedded in international technology competition together with fragmented value creation processes. As a consequence, the interaction between various people and correspondingly the complexity increases. The aforementioned interdependencies and complementarities also provide opportunities in the sense that governmental policies may also make use of indirect interventions.

Finally, to evaluate any technology field and thus to derive an efficient technology policy, it is necessary to have valid data. The underlying indicators in the case of service robots at the industry level or at the level of property rights protection (patents; and related to service, also trademarks) have to be reliable. Since service robots are still an emerging technology field, an efficient innovation policy has to deal with these challenges. This is especially important to avoid governance errors that are due to incomplete or wrong data. One might conclude that any occasional focus on pure technology policy is not up to date. To disentangle the various relationships, interactions and underlying mechanisms still requires a lot of further research.

## Footnotes

1 In doing so, the technology intensity increases both in industry (i.e. in the context of technology development) and services (i.e. technology application).

2 Detailed information on service robots for both professional and private use can be found at www.ifr.org or www.worldrobotics.org/.

3 In 2011, the sales value of professional service robots was almost six times higher than the sales value of service robots for personal and domestic use (IFR press release, September 2012).

4 Source: data based on IFR press release, September 2012.

5 A recent study of the German Federal Ministry of Education and Research (BMBF) provides detailed insights in even more application areas of service robots and gives an estimation of their profitability (Hägele et al. 2011).

6 An internationally utilized concordance table for various technology fields that emerged during the last several decades can be found at the world intellectual property organization’s web page at www.wipo.int/ipstats/en/statistics/technology_concordance.htmla last update August 2011.

7 How these issues are dealt with in the case of emergent nanotechnologies can be seen by Schnorr-Bäcker (2009) w.r.t. national accounts or Mogoutov and Kahane (2007) concerning the identification of patents in international patent databases.

8 Compare Schmoch (2007) who disentangles the underlying effects for the case of industrial robots and already provides some insights concerning service robots.

## References

[CR1] Bresnahan TF, Hall BH, Rosenberg N (2010). General purpose technologies. Handbook of economics of innovation.

[CR2] Chesbrough H, Spohrer J (2006). A research manifesto for service science. Commun ACM.

[CR3] Gehrke B, Rammer C, Frietsch R, Neuhäusler P, Leidmann M (2010) Listen wissens- und technologieintensiver Wirtschaftszweige. Zwischenbericht zu den NIW/ISI/ZEW-Listen 2010/2011, NIW/ISI/ZEW. Studien zum deutschen Innovationssystem 19-2010

[CR4] Grossman GM, Helpman E (1991). Innovation and growth in the global economy.

[CR5] Hägele M, Blümlein N, Kleine, O (2011) Wirtschaftlichkeitsanalysen neuartiger Servicerobotik-Anwendungen und ihre Bedeutung für die Robotik-Entwicklung. Eine Analyse der Fraunhofer Institute IPA und ISI im Auftrag des BMBF, Fraunhofer Gesellschaft

[CR6] Maleri R, Frietzsche U (2008). Grundlagen der Dienstleistungsproduktion.

[CR7] Mogoutov A, Kahane B (2007). Data search strategy for science and technology emergence: a scalable and evolutionary query for nanotechnology tracking. Res Policy.

[CR8] Schmoch U (2007). Double-boom cycle and the comeback of science-push and market-pull. Res Policy.

[CR9] Schnorr-Bäcker S (2009). Nanotechnologie in der amtlichen Statistik. Statistisches Bundesamt, Wirtschaft und Statistik.

